# Comparative Evaluation of Gingival Health Among Children With Stainless Steel Crowns and Stainless Steel Bands: A Split-Mouth Randomized Controlled Trial

**DOI:** 10.7759/cureus.60473

**Published:** 2024-05-16

**Authors:** Ayesha Fathima, Ganesh Jeevanandan

**Affiliations:** 1 Pediatric and Preventive Dentistry, Saveetha Dental College and Hospitals, Saveetha Institute of Medical and Technical Sciences, Saveetha University, Chennai, IND

**Keywords:** pediatric dentistry, stainless steel crowns, space maintainers, gingival health, band and loop

## Abstract

Introduction

Putting in stainless steel crowns as a post-endodontic restoration and space maintainers as a post-extraction appliance to maintain the space for the eruption of underlying teeth in case of early loss in children are some of the most commonly practised procedures that pediatric dentists undertake in their day-to-day lives. Maintaining good oral hygiene for better gingival health is important. If it is not taken care of, gradual destruction of supporting soft and hard tissues of the teeth occurs. There were numerous studies conducted over the past few years, but no split-mouth study compares the crowns and bands. Hence, this study compares the gingival health between stainless steel crowns and band and loop space maintainers.

Materials and methods

This split-mouth randomised controlled trial included 31 children aged between four and nine years who had stainless steel crowns on one side and a band and loop space maintainer on the other side of the mandibular arch. The split-mouth study was carried out to minimise the outcome bias as oral hygiene practices differ from one individual to another. Presence/absence of bleeding on probing (BOP) and the Gingival Index (GI) using the Loe and Silness GI were assessed at baseline and at six months. Data was entered in an Excel sheet (Microsoft Corporation, Redmond, Washington, United States) and analysed in IBM SPSS Statistics for Windows, Version 23, (Released 2015; IBM Corp., Armonk, New York, United States). The significance level was fixed as 5% (α = 0.05). The Shapiro-Wilk test was used to assess the normality of parameters of gingival health. The parameters are described in terms of mean, standard deviation, frequency and percentages. Intragroup analysis was done using Friedman tests across the timelines. Intergroup analysis using Mann-Whitney U tests was done between the groups at different timelines.

Results

At the beginning of the study, 46 children (22 girls and 24 boys) were enrolled considering the dropout. However, 15 children did not attend the follow-up review, resulting in a loss to follow-up. Consequently, only 31 children, each with a band and loop space maintainer and a stainless steel crown, were included for the final interpretation of the results in this study. At one month, both BOP and GI were significantly different (p<0.05) between the stainless steel crown and stainless steel band where the crown showed better gingival health and absence of bleeding than the band and loop. At three months and six months, gingival health improved in both groups, but there was no significant difference between the groups. The Friedman test revealed that both the stainless steel crown and stainless steel band groups had a significant difference at six months from baseline. Mann-Whitney tests were done to analyse the difference in parameters at baseline and at six months between both groups. There was no significant difference in the baseline in the parameters between the groups.

Conclusion

Within the limitations of the study, this study concludes that the gingival health based on BOP and GI shows a significant difference across the timeline within the groups, namely, stainless steel crown and stainless steel band, but no significant difference between the groups at various timelines.

## Introduction

Early exfoliation of primary teeth most commonly due to non-restorable dental caries or trauma can cause loss of space, arch discrepancy, crowding, and malocclusion in a growing child. The use of appropriate space maintainers may prevent later complications such as ectopic eruption, impaction of successor teeth, and malocclusion [1]. The management for preventing such complications is to use an appropriate space maintainer that is durable, cost-efficient, and clinically effective until the eruption of its underlying permanent teeth [2]. A space maintainer is an appliance used in children to maintain the mesiodistal width in a dental arch when premature loss of primary teeth occurs [3]. It is advised to utilize space maintainers appropriately to preserve space until permanent teeth emerge to prevent space loss and lessen its effects [4]. 

It is preferable to replace a space maintainer in the first month following tooth loss because studies have shown that space loss happens in the first three to six months following tooth loss [4,5]. Depending on the dental developmental stage of the kid, the dental arch involved, the number of teeth involved, the placement of the teeth, and the type of primary teeth involved, several appliances can be utilized as space maintainers. It is advised to use either fixed or removable space maintainers to prevent malocclusion, which is caused by the early loss of deciduous teeth [6]. 

Removable space maintainers are great since they're simple to clean and help kids practice better oral hygiene, but they can also be misplaced, worn out, or damaged if not handled appropriately. In addition to this, they won't work well if not used correctly. Retention and acceptance are two further issues with these kinds of space maintainers, particularly in this vulnerable age group. Fixed appliances were the treatment of choice predominantly. The band and loop space maintainers are the most commonly used fixed space maintainers in pediatric dentistry. Also, they are easy to fabricate and economical [8]. However, these space maintainers are used when there is a unilateral loss of primary molars [9]. 

Extensive carious lesions involving more than one tooth surface in the deciduous dentition require a complete tooth covering restoration. Stainless steel crowns (SSCs) were the choice of full coronal restoration, as they were easily available as preformed, pretrimmed, and pre-contoured crowns with a wide range of sizes and with proven clinical efficiency [10]. Many research works have examined the gingival condition of primary molars that have been restored using SSCs. Healthy gingiva and reduced plaque accumulation were associated with well-to-moderately integrating crowns as well as well-contoured edges [11,12].

Though space maintainers and crowns are different entities with their own set of indications, our hypothesis was whether band and loop space maintainers due to their open-faced nature on the occlusal surface of the teeth would accumulate more plaque retention resulting in inflammation compared to SSCs and how are their variations at various timelines. 

Local factors play a significant role in the development of gingivitis and plaque retention. Reports suggest that gingival infections stemming from fixed orthodontic appliances like brackets and bands can lead to oral hygiene issues, including irritation, bleeding, and increased pocket depth [13]. Exposed cement and the design of orthodontic wire can cause mechanical or chemical irritation, while their structure facilitates plaque accumulation, particularly around the gingival margin, making cleaning with toothbrushes and floss more challenging [14].

Studies indicate that orthodontic appliances promote plaque buildup and gingivitis. Orthodontic bands and brackets contribute to plaque proliferation, potentially leading to various periodontal issues during treatment, ranging from minor attachment loss to irreversible changes [15]. Given that patients receiving space maintainers tend to be younger and may struggle with oral hygiene compliance, the consequences of plaque accumulation could be more severe in this demography. This study aims to compare gingival health between stainless steel bands (SSBs) and SSCs.

## Materials and methods

Study setting and population

This prospective split-mouth trial was conducted from December 2021 to October 2022 at the Outpatient Department of Pediatric and Preventive Dentistry at Saveetha Dental College and Hospitals in Chennai, India. Thirteen girls and eighteen boys between the ages of five and nine participated in this study. Children who required endodontic management followed by an SSC on one side and extraction of a primary molar followed by band and loop space maintainer on the other side of the maxillary arch were included in the clinical trial. 

Ethical clearance

Before the study began, ethical approval was acquired by the Scientific Review Board of Saveetha Dental College and Hospitals (SRB/SDC/PEDO-2104/21/142). The study adhered to the principles outlined in the Declaration of Helsinki regarding the ethical conduct of research involving human subjects. Measures were taken to ensure the well-being and safety of the participants throughout the study. Participants and parents on their behalf were provided with information about the study clearly and understandably, and they were allowed to ask questions and seek clarification before obtaining consent. Confidentiality of the participant's personal information and data was maintained, with access limited to only those directly involved in the research. Participants were informed of their right to withdraw from the study at any time without facing any consequences or loss of benefits.

Inclusion criteria

Children between four and nine years who required pulpal therapy followed by an SSC on one side and grossly decayed, unrestorable primary molar that required extraction followed by space maintenance therapy on the maxillary arch were included in the study. No decay/missing teeth were present in both arches. Patients who were positive and definitely positive on Frankl's behaviour rating scale were included to ensure homogeneity within the sample size and enhance the validity and reliability of the study findings. Children not having any malocclusion such as crowding, spacing, and crossbite, which have a direct influence on gingival health, was another inclusion criterion. Only those children were included whose parents gave consent for the study.

Exclusion criteria

Systemically/medically compromised children, children who were allergic to chromium or nickel, and children whose parents would not be able to meet up for regular check-ups/follow-ups were excluded.

Sampling

The sample size determination was guided by the study conducted by Hosseinipour et al. [11], which utilized a significance level (α) of 0.05% and achieved a statistical power of 95% with an effect size of 0.636. Utilizing G*Power software (Heinrich Heine University Düsseldorf, Düsseldorf, Germany), the estimated sample size was calculated to be 31 participants. Considering a potential dropout rate of 40%, the initial sample size was increased to 46 participants at the outset of the study. This sample size was deemed appropriate to meet the study's objectives and ensure sufficient statistical power to detect meaningful effects in this study. Participants who failed to attend follow-up appointments were excluded from the final data analysis conducted for statistical purposes in the study.

Survey instruments

The study employed two instruments to evaluate the gingival health of the patients: bleeding on probing (BOP) and the Gingival Index (GI). BOP and GI were examined on the teeth with SSCs and the teeth where the SSBs of the space maintainers were placed. We conducted rigorous validation procedures and assessed their reliability before implementing them in the study, ensuring the accuracy and consistency of our data collection methods.

BOP was examined using a Michigan probe with Williams marking with a 0.4 mm diameter kept parallel to the long axis of the tooth [16,17]. The probe was made to run through the sulcus with a pressure ranging between 20 and 25 grams. If bleeding was observed within 10 to 15 seconds of probing, it was recorded as present. If not, BOP was marked absent [18].

The measurement of the GI adheres to the criteria outlined in the GI system developed by Loe and Silness. The buccal, lingual, mesial and distal gingival regions of the tooth were given a score ranging from 0 to 3, known as the GI score for those teeth. The GI for the tooth is calculated by adding the scores from the four zones of the tooth and dividing the result by 4 (0 = normal gingiva; 1 = mild inflammation: a slight change in colour and slight oedema but no BOP; 2 = moderate inflammation: redness, oedema and glazing, and BOP; 3 = severe inflammation: marked redness and oedema, ulceration with a tendency to spontaneous bleeding) [19].

The child along with the parent was educated and motivated to use fluoridated toothpaste with soft bristles brush at least once a day. 

Data collection

**Figure 1:**
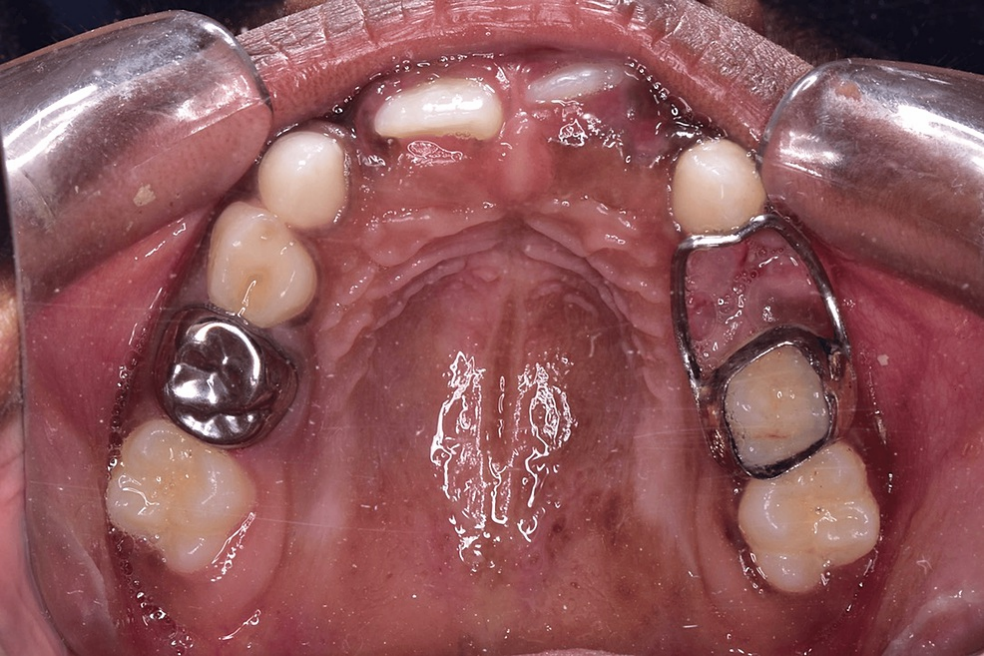
Participant with stainless steel crown placed on one side and band and loop space maintainer on the other side of the arch

The fabrication of all space maintainers was consistently performed by a trained dental technician throughout the study. Both SSCs and space maintainers were delivered and cemented on the same day for the participants (Figure 1). The children underwent assessment preoperatively for BOP and GI on the day the SSCs and SSBs were placed on opposite sides. Follow-up clinical examinations at baseline, one, three, and six months were conducted by the same postgraduate training dentist, who had undergone calibration to ensure reproducibility before the study. 

Statistical analysis

Data was entered in a Microsoft Excel spreadsheet (Microsoft Corporation, Redmond, Washington, United States) and analyzed using IBM SPSS Statistics for Windows, Version 26, (Released 2019; IBM Corp., Armonk, New York, United States). The significance level was fixed as 5% (α = 0.05). The Shapiro-Wilk test was used to assess the normality of parameters of gingival health. The parameters are described in terms of mean, standard deviation, frequency and percentages.

Data was analysed by descriptive statistics, which included frequency, percentages, mean and standard deviation with a 95% confidence interval. Intragroup analysis using Friedman tests to assess the differences in BOP and GI across the timelines within the groups was done. Intergroup analysis using Mann-Whitney U tests to assess the differences between the groups at baseline, one month, three months, and six months was done.

## Results

Descriptive data

At the beginning of the study, 46 children (22 girls and 24 boys) were enrolled. However, 15 children did not attend the follow-up review, resulting in a loss to follow-up. Consequently, only 31 children, each with a band and loop space maintainer and an SSC, were included for the final interpretation of the results in this study. The study population consisted of 31 children who visited for regular appointments till six months of follow-ups. The mean age of the children was 6.143 ± 0.332 years. Out of the 31 children, 13 were girls and 18 were boys. BOP and GI were assessed for both SSCs and SSBs. The frequency and percentage of BOP and GI for SSCs and SSBs were evaluated at baseline, one month, three months, and six months (Tables 1, 2). It was observed that there was an inclination toward a positive BOP in the first month in both groups. In the third month, there was an increase in scoring in positive BOP for SSCs, and it remained the same for the third month in the SSB group. An abrupt decline was observed in both groups in the sixth month. Similarly, for the GI, there was a spike in mild and moderate gingivitis in the first month and it gradually decreased over the sixth month follow-up.

**Table 1:** Distribution of bleeding on probing (BOP) between stainless steel crowns and stainless steel bands at different points in the timeline.

Group	Timeline	BOP negative n (%)	BOP positive n (%)
Stainless steel crown	Baseline	28 (90.3)	3 (3.7)
	One month	15 (48.4)	16 (51.6)
	Three months	1 (3.2)	30 (96.8)
	Six months	22 (71)	9 (29)
Stainless steel band	Baseline	28 (90.3)	3 (3.7)
	One month	5 (16.1)	26 (83.9)
	Three months	5 (16.1)	26 (83.9)
	Six months	23 (74.2)	8 (25.8)

**Table 2:** Distribution of the Gingival Index between stainless steel crowns and stainless steel bands at different points in the timeline.

Groups	Timeline	No gingival inflammation n (%)	Mild gingivitis n (%)	Moderate gingivitis n (%)
Stainless steel crown	Baseline	15 (48.4)	14 (45.4)	2 (6.5)
	One month	0	18 (58.1)	13 (41.9)
	Three month	20 (64.5)	4 (12.9)	7 (22.6)
	Six months	12 (38.7)	19 (61.3)	0
Stainless steel band	Baseline	15 (48.4)	14 (45.4)	2 (6.5)
	One month	0	18 (58.1)	13 (41.9)
	Three months	12 (38.7)	4 (12.9)	15 (48.4)
	Six months	11 (35.5)	12 (38.7)	8 (25.8)

Intragroup analysis using Friedman tests was done to analyse the change in the parameters from baseline to six months within the group. Both BOP and GI increased at one month and three months from baseline but gradually decreased at six months. Both crown and band groups had a significant difference at six months from baseline (p<0.05) (Table 3).

**Table 3:** Intragroup analysis showing significant differences in bleeding on probing (BOP) and the Gingival Index (GI) among both groups across the timeline. *p<0.05: statistically significant.

Category	Baseline	One month	Three months	Eight months	Chi-square value	p-value
BOP - stainless steel crown	1.76	2.6	3.5	2.15	45	0.000*
BOP - stainless steel band	1.68	3.16	3.16	2	47.642	0.000*
GI - stainless steel crown	2.15	3.35	2.24	2.05	23.886	0.000*
GI - stainless steel band	1.87	2.98	2.79	1.78	17.589	0.001*

Intergroup analysis using Mann-Whitney tests was done to analyse the difference of parameters at baseline and at six months between the two groups. There was no significant difference in the baseline in both the parameters between the groups. At one month, both BOP and GI were significantly different between SSCs and SSBs (p<0.05) where the SSCs showed better gingival health and absence of bleeding than the SSBs. At three months and six months, gingival health improved in both groups, but there was no significant difference between the groups (Table 4).

**Table 4:** Intergroup analysis between the groups showed the differences in bleeding on probing (BOP) and the Gingival Index (GI) between the groups at different points in the timeline. *p<0.05: statistically significant.

	Group	Mann Whitney U value	p-value
Baseline BOP	Stainless steel crown	480.5	1.000
	Stainless steel band		
One month BOP	Stainless steel crown	325.5	0.007*
	Stainless steel band		
Three months BOP	Stainless steel crown	418.5	0.08
	Stainless steel band		
Six months BOP	Stainless steel crown	465.0	0.778
	Stainless steel band		
Baseline GI	Stainless steel crown	480.5	1.000
	Stainless steel band		
One month GI	Stainless steel crown	340.5	0.029*
	Stainless steel band		
Three months GI	Stainless steel crown	425.5	0.654
	Stainless steel band		
Six months GI	Stainless steel crown	389.0	0.155
	Stainless steel band		

## Discussion

SSCs, also known as chrome steel crowns or preformed metal crowns, are metallic restorations that have shown good long-term retention and significant clinical success in the restoration of larger carious lesions on primary molars. SSCs remain an integral part of pediatric dental care [10]. The band and loop space maintainer, on the other hand, consist of an open-faced SSB placed on an abutment tooth, and a loop is extended from the buccal and lingual/palatal surfaces of the band to the adjacent teeth across the edentulous space. This appliance basically maintains the space created by the early loss of the primary molar until the underlying permanent tooth emerges [20]. Gingival health is a critical aspect of overall oral health, and it can be affected by various factors such as oral hygiene practices, dietary habits, systemic health of an individual, etc. However, this study focuses specifically on understanding the impact of prosthetic devices on gingival health in pediatric patients [21]

SSCs and SSBs have a sharp edge and can penetrate deeper into the gingival sulcus [22]. Thus, the gingiva is more susceptible to traumatization during crown band placement and challenges in maintaining oral hygiene due to their contact with gingival margins and soft tissue strain [23,24]. In this study, the GI and BOP were utilized to evaluate gingival health. The present study portrayed positive BOP and mild to moderate gingivitis in the majority of the first and third months providing us insights to understand that both SSCs and SSBs contribute to the development of oral biofilms by altering the oral cavity's area and surface properties. Additionally, the presence of the crown and space maintainer in the oral cavity has been associated with elevated plaque formation, which then decreases gradually over a six-month period.

Initially, gingival tissues undergo an adaptation period during which they adjust to the presence of prostheses such as SSCs and SSBs, which would be responsible for the changes observed in gingival health within the first month [25,26]. However, the subsequent improvement seen over the following months suggests that the gingival tissues successfully acclimate and adapt to the prosthesis. This is consistent with findings from Arıkan et al., who compared gingival health and periodontal health in fixed and removable space maintainer users and concluded that both types of devices may lead to an increase in oral cavity microorganisms and periodontal index scores [27].

A significant decrease in the time-dependent evaluation of the means of plaque and gingival indexes was observed in previous studies in which pediatric patients using fixed and removable appliances were followed up for six months. Significant increases were observed in the plaque index, GI, and gingival bleeding index values. These results showed similarities to our findings at three three-month recall period points [28,29]. Consistent with our results, in a study conducted by Nikhil et al., the zirconia crowns and SSCs in primary molars were evaluated and compared. It was determined that, for both SSCs and zirconia crowns, there was a statistically significant increase in the GI and Oral Hygiene Index (OHI) scores after three months, followed by a decline in scores at six and nine months. After six months, there was no significant difference in gingival health between the two groups [30]. Hence, it can be inferred that any form of prosthesis altering the normal oral environment is not absolutely gingival-friendly. During the use of SSCs or SSBs, it is crucial to closely monitor patients and educate them about the potential risks associated with the use. Patients must be reminded to pay special attention to their oral hygiene practices.

The results revealed notable changes in both SSC and SSB usage over the initial six-month period, with a temporary decline in gingival health observed within the first month. However, it is significant to note that gingival health gradually improved thereafter, reaching a better state by the end of the study period. Importantly, no discernible difference in the impact on gingival health was observed between SSCs and SSBs. These findings emphasize the importance of regularly assessing gingival health in children with space maintainers, SSCs, or any other form of dental prosthesis, particularly during the initial stages of use. Future studies could further investigate the long-term consequences and potential factors influencing gingival health.

The present study has its limitations. Only two groups (crown and band) were assessed in the study. There was no inclusion of removable space maintainers. There was only a comparison of one material, stainless steel, and no comparison of any other material, such as zirconia, was included. Only BOP and GI were used when many other variables like the United States Public Health Service (USPHS) criteria could have been assessed, which would have added more credibility to the study. The study primarily focused on short-term impacts and adaptation periods; long-term effects of space maintainers on gingival health and overall oral hygiene beyond the initial adaptation phase would have added more credibility to the study. There should be more studies conducted to include various types of space maintainers.

## Conclusions

Within the limitations of the study, we can conclude that the gingival health based on BOP and GI shows a significant difference across the timeline within the two groups, namely, SSC and SSB, but no significant difference between the groups at various points in the timeline. Also, this study highlighted the necessity and importance of routinely assessing gingival health in children wearing any prosthesis, particularly in the early stages of use.
